# SMS-reminder for vaccination in Africa: research from published, unpublished and grey literature

**DOI:** 10.11604/pamj.supp.2017.27.3.12115

**Published:** 2017-06-22

**Authors:** Kasidet Manakongtreecheep

**Affiliations:** 1 Yale University, New Haven, USA

**Keywords:** Immunization, coverage, vaccination, schedule, eradication, SMS, reminder

## Abstract

Immunization for children against vaccine-preventable diseases is one of the most important health intervention method in the world, both in terms of its health impact and cost-effectiveness. Through EPI and various other programs such as the Decades of Vaccines, immunization has been improving the health of children around the world. However, this progress falls short of global immunization targets of the Global Vaccine Action Plan (GVAP). Furthermore, the African region still lags behind in immunization, and suffers from a high proportion of vaccine preventable diseases as a result. Reminders and recall for vaccination have been shown to improve health care-seeking behaviours, and have been recommended for application in routine and supplemental measles immunization activities. With mobile phones becoming more accessible in Africa, SMS vaccine reminder system has been proposed as a convenient and easily scalable way to inform caregivers of the disease and the importance of immunization, to address any concerns related to immunization safety, and to remind them of vaccination schedules and campaigns. There have been 6 published articles and 1 unpublished article on the effect of SMS reminder system for immunization in Africa. The studies done has shown that SMS vaccination reminder has led to improvements in vaccination uptakes in various metrics, whether is through the increase in vaccination coverage, decrease in dropout rates, increase in completion rate, or decrease in delay for vaccination.

## Introduction

Immunization for children against vaccine-preventable diseases is one of the most important health intervention method in the world, both in terms of its health impact and cost-effectiveness. Proper immunization is not only important for the vaccinated individuals, but also for the general population [1]. In 1974, the World Health Assembly launched the Expanded Program on Immunization (EPI), with an aim to increase routine immunization rate around the world [2]. Through EPI and various other programs such as the Decades of Vaccines, immunization has been improving the health of children around the world, and the number of children who did not receive routine vaccinations has dropped to an estimated 19.4 million, down from 33.8 million in 2000 [3]. Immunization coverage for three doses of Hepatitis B vaccine has increased from 29% in 2000 to 84% in 2015 [4]. However, this progress falls short of global immunization targets of the Global Vaccine Action Plan (GVAP) of 90% or more DTP3 vaccination coverage at the national level and 80% or more in all districts in all countries by 2015. Of the 6 targets of GVAP, 5 of them are off-track, and would not be achieved if no new strategies were found [5].

Despite 126 countries achieving the 90% vaccination coverage target for DTP, with high coverage achieved in the Americas, Europe, the Western Pacific, and the Eastern Mediterranean Ocean, the African region still lags behind in immunization, and suffers from a high proportion of vaccine preventable diseases as a result. About 1.5 million children around the world die each year from vaccine-preventable deaths, with Africa suffering the most [6]. The 2001-2005 African Regional Strategic Plan of the Expanded Programme on Immunization, with a goal for 80% of the countries in Africa to reach 80% immunization coverage by 2005, was not achieved [7]. In 2016, the immunization coverage for Africa region is 80%, lowest of any region in the world [9]. In 2014, five of the six countries with less than 50% DTP3 coverages are African countries. The MCV1 coverage in Africa is 74%, the lowest amongst the regions and much lower than the global coverage of 85% [8]. Furthermore, 1 in every 5 in Africa do not have access to life-saving vaccines [9]. New innovative ideas and interventions must be seriously considered in order for Africa to reach the GVAP goal and save the lives of millions of children.

Reminders and recall for vaccination have been shown to improve health care-seeking behaviours, and have been recommended for application in routine and supplemental measles immunization activities [10]. In Cochrane Review, 47 studies on vaccine reminder in developed countries were evaluated, and found that all reminder systems increased the number of people vaccinated, whether the reminder were for people due or overdue for vaccinations. There are however, several barriers to the incorporation of reminder and recall system. One of the reasons is that health providers do not perceive the studies as applicable to their own practices or applied to their settings. Different vaccination criteria in the studies also makes it difficult to extrapolate the result with one vaccination schedule to real-world implementation with a different vaccination schedule. Another important reason is that many providers lack the computerized system to track the patients’ immunization status [11]. However, with the recent advances in technology and accessibility, reminder and recall systems are being used more and more in both developed and developing countries. Mobile phone, specifically SMS, is being considered as a tool for vaccination reminder and recall.

### SMS reminder system for immunization in Africa

Mobile phones are becoming more accessible in Africa, as the costs of services are getting cheaper [12]. Ownership has been surging rapidly, with some countries achieving more than ten-fold increase in ownership from 2002 to 2014. In 2015, there are already more than 635million mobile subscriptions in sub-Saharan Africa, and it is predicted to rise to about 930million by late 2019 [13]. Even in rural communities, non-smart phones are being used as a major tool to access information, mostly through SMS.

One of the biggest reasons for missed vaccination in children in Africa is the lack of communication between mothers and health workers, which could lead to a lack of knowledge on vaccination, or lack of proper service or vaccines on the day of vaccination [14]. Another risk factor associated with incomplete immunization is the forgetfulness of the mother on the day of immunization [15]. In a study, a survey found that 98% of respondents thought their children were immunized, but only 50% were immunized according to the recommendations. Furthermore, the survey found that “only half of parents with two year-olds and one-third of parents with seven-year-olds reported their child had received all of the recommended doses” of routine immunization [16]. Parents objection, disagreement or concern about immunization safety is also considered a major reason for missed or partial immunization in children [17].

SMS can be used as a convenient and easily scalable way to inform caregivers of the disease and the importance of immunization, to address any concerns related to immunization safety, and to remind them of vaccination schedules and campaigns. Furthermore, SMS can be sent to inform mothers of any issues at the health facility, such as stock-outs and long waiting time, in order to inform mothers to visit a different health facility or come on another day. SMS reminders have the potential to help increase vaccination coverage, as well as to increase the public’s positive image of vaccination activity.

Overall, more than 40 published studies in Cochrane review from different regions have proven the effectiveness of vaccine reminders. In Africa, SMS vaccine reminder system has been done in a small scale in a few countries. In Mozambique, about 35,000 children in the northern province of Nampula have been registered with the mVaccination pilot programme, launched in 2015 by GSK and Vodafone, with funding from GAVI and USAID. In December 2015, the service was expanded from17 to 76 healthcare facilities [18]. In Benin, 90 health facilities in two states are using an electronic registration and vaccine reminder system through a tablet application, called VaxTrac, with funding support from BMGF [19]. With the surge in mobile phone ownership in developing countries, SMS provide the obvious solution to missed vaccinations in these countries.

## Methods

Although there has been no full-scale implementation of SMS vaccine reminder system in Africa at a national level, many studies and small-scale interventions have been done in the African region in the past few years (Table 1). This supplement summarizes the 5 published articles on the effect of SMS-reminder system for immunization in Africa, 1 to-be-published article on the effect of SMS-reminder system for immunization in Africa, and 1 unpublished article on SMS-reminder system for Vitamin A supplement. The articles included were chosen with the criteria that all studies included (1) an intervention in the form of a reminder given through SMS; and (2) the effect of the intervention, assessed quantitatively in the form of improvement in vaccination; there is one exception, being the study on Vitamin A supplement uptake, which is not considered a vaccine. The summary was put into four tables listing: (1) basic information, (2) information on methods of the research, (3) information on results, (4) information on the SMS software and additional protocol on the SMS system. It is hoped that the supplement can help inform health practices on the effectiveness of this media platform in reminder and recall system for immunization, especially in African setting.

**Table 1:** Most important information from SMS-reminder for vaccination research findings in Africa

Country	Kenya		Nigeria		Zimbabwe	Burkina Faso	Senegal
Author	Gibson et al.^i^	Haji et al.	Brown et al.^iI^	Eze, Adeleye	Bangure et al.	Schlumberger et al.	Thiaw et al.^iii^
Year of Publication	2017 forthcoming	2016	2016	2015	2015	2015	2013
Impact of SMS	Vaccination coverage increased by 4% for SMS only, and by 8% for SMS+200KES CCT^iv^	Dropout rates decreased by 13%	RI completion rate increased by 47.3%	Timely completion rate increased by 8.7%	Vaccination coverage increased by 15% at 6 wks, by 16% at 10wks and by 20% at 14 wks	Immunization completeness for pentavalent 3rd dose increased by 18%	Vitamin A supplement coverage increased by 58.9% from baseline for intervention districts
Experiment Type	Cluster randomized controlled trial	Intervention evaluation	Group randomized control trial	Randomized control trial	Randomized control trial	Randomized control trial	Randomized control trial
Total Sample Size	2018 from 152 villages	1116 from 3 districts	595 from 4 LGAs^v^	905 from 8 health facilities	304 from multiple clinics	523 from 1 health center	1137 from 6 districts

i Forthcoming article (2017) at Lancet Global Health

ii No full article access, information gathered only from abstract

iii Unpublished report by the Helen Keller Foundation

iv Conditional Cash Transfer

v Local Government Areas

## Results

There have been 5 published articles and 2 unpublished article on the effect of SMS-reminder system for immunization in Africa. Of those, 2 are done in Kenya and 2 are done in Nigeria. All studies have been shown to either improve immunization coverage, or reduce dropout rates for vaccination. All but one article used randomized control trial as the type of the study, with one intervention evaluation study in Kenya. All but one article used the reminder for vaccination, with one study using the reminder for Vitamin A administration. The primary outcome measure used in most studies are either the completion rate of the whole vaccine set, or the completion rate for pentavalent vaccine 3rd dose or for DPT3, with one study using the decrease in dropout rates as the primary outcome measure. Although there are control results in all studies, only 3 studies contain baseline measure (Table 1).

### Additional intervention to SMS reminder

While 3 studies only focus on SMS reminder system as a sole intervention, with no reminder system as the control, 4 studies added other forms of intervention alongside SMS reminder system, either together or as a combined intervention, or as another monitored intervention in itself. A study uses conditional cash transfer in addition to SMS reminder, alongside the normal SMS reminder [20]. Another study used Primary Health Care Immunization Providers’ Training (PHCIPT) as an additional intervention, tested together with SMS reminder and separately [21]. A study used sticker reminder separately to compare to SMS reminder and control [22]. The study on reminder for Vitamin A also had an updated health card as an intervention [23] (Table 2).

**Table 2:** Additional information from SMS-Reminder for vaccination research findings in Africa: methods

Country	Kenya		Nigeria		Zimbabwe	Burkina Faso	Senegal
Author	Gibson et al.	Haji et al.	Brown et al.	Eze, Adeleye	Bangure et al.	Schlumberger et al.	Thiaw et al.
Eligibility of Participants	Children aged <5 weeks who had not received 1st pentavalent vaccine	Children aged <12 months presenting for their 1st pentavalent vaccine	Children aged 0-12 wks	Children aged 0-5 years brought for routine immunization for the first or second time	Newborns of women who delivered and were residents of Kadoma City	Children at the time of their birth or at first EPI session	Children aged 2-5 months
Size of Control Group	489	372	N/A	453	152	268	582
Intervention Type (Size)	SMS only (n=476), SMS+75 KES (n=562), SMS+200KES (n=491)	SMS (n=372), sticker (n=372)	SMS (n=N/A), PHCIPT (n=N/A), SMS+PHCIPT^i^ (n=N/A)	SMS (n=452)	SMS (n=152)	SMS (n=253)	SMS+Vitamin A Supplement health card (n=555)
Trial Period for Each Child	12 months	16 wks	N/A	12wks or 18 wks^ii^	14 wks	12 months	N/A

i i. Primary Health Care Immunization Providers’ Training

ii 12 wks trial period for children brought for routine immunization for the second time (at 6 wks) and 18 wks trial period for children brought for routine immunization for the first time (at 0 wks).

The study in Kenya by Gibson et al., which uses conditional cash transfer in addition to SMS reminder intervention, shows that better immunization coverage can be achieved with additional cash incentives. This is however, only achieved with certain amount of money, as KSH 75 cash incentive + SMS intervention did not increase the coverage from the normal SMS intervention, but KSH 200 cash incentive + intervention increased the coverage by 4% from normal SMS intervention and by 8% from the control coverage. Another study that pairs SMS with additional intervention was done in Nigeria by Brown et al., with PHCIPT (Primary Health Care Immunization Providers' Training) paired with SMS reminder. The study however, found that the group using SMS alone actually had a slightly better routine immunization (RI) completion rate (98.6%) as compared to SMS+PHCIPT (97.3%), and a much better immunization rate than the group with PHCIPT alone (70%). In the 2016 study in Kenya by Haji et al, sticker reminder intervention was also tested, and found that it did not lead to a big decrease in dropout rate when compared to the control (16% dropout rate in sticker reminder and 17% in control). SMS reminder however, showed much higher decrease in dropout rate, from 17% in control to only 4% in SMS reminder intervention group (Table 3).

**Table 3:** Additional information from SMS-Reminder for vaccination research findings in Africa: results

Country	Kenya		Nigeria		Zimbabwe	Burkina Faso	Senegal
Author	Gibson et al.	Haji et al.	Brown et al.	Eze, Adeleye	Bangure et al.	Schlumberger et al.	Thiaw et al.
Primary Outcome Measure	Immunization coverage after 12 months (FIC)	Dropout rate for pentavalent 3rd dose	RI completion rate at 12 months of age	DPT3 timely completion rate	Immunization coverage at 6 wks, 10 wks and 14 wks	Immunization completeness for pentavalent 3rd dose + OPV + rotavirus	Vitamin A supplement coverage
Outcome Result Based on Primary Measure (intervention group)	86% (SMS only), 86% (SMS+75 KES), 90% (SMS+200KES)	4% (SMS), 16% (sticker)	98.6% (SMS), 70% (PHCIPT), 97.3% (SMS+PHCIPT)	69% (SMS)	97% at 6 wks (SMS), 96% at 10 wks (SMS), 95% at 14 wks (SMS)	73.3% for OPV1 (SMS), 71.3% for OPV2 (SMS), 60.3% for OPV3 (SMS)	67.7% (SMS)
Outcome Result Based on Primary Measure (control)	82%	17%	57.30%	60%	82% at 6 wks, 80% at 10 wks, 75% at 14 wks	55.7% for OPV1, 53.6% for OPV2, 42.3% for OPV3	6%
Baseline	N/A	13%	N/A	58%	74% at 6 wks, 84% at 10 wks, 74% at 14 wks	N/A	8.8% (intervention group) 3% (control group)
Confidence Interval or p-value	p=0.045 (SMS only), p<0.0001 (SMS+75 KES), p<0.0001 (SMS+200KES)	CI 0.04-0.8 (SMS)	CI 1.50-1.98 (SMS), CI 1.03-1.45(PHCIPT), CI 1.47-1.95 (SMS+PHCIPT)	p=0.009, CI 1.103-1.955 (SMS)	p<0.001 (SMS)	p<0.001 for OPV1 (SMS), p<0.001 for OPV2 (SMS),	N/A

### SMS reminder software and protocol

Of the 7 studies on SMS-reminder for vaccination, 3 studies disclosed the name of the software, while one study stated that it had used “Internet-based web-to-SMS”. Only one study used UNICEF’s open-sourced software called Rapid SMS, which was developed for sending automatic mass SMS messages. Of the studies that listed their SMS schedules, all but one study scheduled their SMS to remind the parents days before the vaccination date, and all but one sent multiple SMSs leading up to the date. Reminder/Recall system, which is a system that sends a message after the schedule to remind and follow up the mother after the time was due, was only used in two studies. The study in Zimbabwe contained a phone call follow-up and the study in Senegal contained a household visit as a follow-up instead. The cost of the SMS message (per one benefactor) was only shown in 3 studies. The study in Zimbabwe showed the highest cost per benefactor of 0.99USD while the study in Nigeria showed the lowest cost of 0.09 USD (Table 4).

**Table 4:** Additional information from SMS-Reminder for vaccination research findings in Africa: SMS software and protocol

Country	Kenya		Nigeria		Zimbabwe	Burkina Faso	Senegal
Author	Gibson et al.	Haji et al.	Brown et al.	Eze, Adeleye	Bangure et al.	Schlumberger et al.	Thiaw et al.
SMS Reminder Software	Rapid SMS	Sematime	N/A	“Internet-based web-to-SMS”	N/A	N/A	Telerivet
Cost per Benefactor	N/A	0.27 USD	N/A	0.09 USD	0.99 USD	N/A	N/A
SMS Schedules	6 wks, 10 wks, 14 wks, 9 months	10 wks, 14 wks	N/A	6 wks, 10 wks, 14 wks	6 wks, 10 wks, 14 wks	2 months, 3 months, 4 months	6 months
SMS per Schedule	3 days before and 1 day before scheduled vaccination	2 days before and on the day of the scheduled vaccination	R/R^i^: N/A	R/R: 1 day before, 1 day after scheduled vaccination	7 days before, 3 days before, 1 day before schedule vaccination	N/A	on the day of scheduled vaccination
SMS follow up after vaccination date	No	No	Yes	Yes	No, but contain follow up by phone call	No	No, but contain household follow up
Message Content	“Tell Mama <BABY’S FIRST NAME>> that <VACCINE NAME> vaccine is due this week. You get KES X if Baby vaccinated in next 2 weeks. <MOTIVATION- AL MESSAGE>”	N/A	N/A	“Dear client, your child is due for his/her next dose of vaccines tomorrow Tuesday 20/7/10. Kindly bring your child to Hospital X for vaccination at 8am. Please come with immunization card. Thank you.”	“Immunization protects your child against killer diseases such as polio, whooping cough,.. You are reminded that the vaccination appointment will be due in X days’ time from today.”	N/A	N/A

i Reminder and Recall system, a system that sends messages to children that missed their vaccination schedule

### Additional information

In the Zimbabwe study by Bangure et al., on top of the vaccination coverage, the association between receiving short message services and delay in receiving vaccinations was also measured. The study found that the SMS reminder intervention group were 75% less likely to delay in having their children immunized than the control group. This means that the age of the children in the non-intervention group will be older than the correct age of immunization, and they can be exposed to these vaccine preventable diseases. The study also asked participants about their willingness to accept the SMS system, and found that majority of the mothers hold positive attitude towards the SMS system [24]. This result further confirmed an earlier study in 2012 on mothers’ willingness to SMS reminder for vaccination, which showed 77% of mothers willing to use the SMS reminder service [25].

In the Kenya study by Haji et al., the delay for vaccination was also found in the non-intervention and the sticker group, whereas the SMS intervention group showed no significant delay. Multivariate analysis was also done to find factors related to missed immunization. The study found that low maternal education (below secondary education) and long distance from health facility (> 5km from health facility) increase the odds of missing vaccinations. On top of the dropout rates, Haji et al. also surveyed for the reasons for dropping out, and founded that 35% of the dropped vaccinations were due to vaccination taken at a different facility, while 30% were due to the caretaker and children being out of town. Forgetting the date were 15% of the cause of missed vaccination, which shows that a significant amount of people who dropped out of the vaccination program could potentially be saved by a vaccine reminder system [26].

In the study in Nigeria by Eze et al., the cost effectiveness of SMS reminders was also calculated, with comparisons made with the use of Junior Community Health Extension Workers (CHEWs) for functional home visits. The results showed SMS reminder as being more cost-effective by 0.25 USD per child. In this study, the reasons for refusal to take part and register for SMS reminders was also surveyed. Result shows that self-confidence in not forgetting appointments (61% of refusals) and the fear of giving out phone numbers (28.8% of refusals) were the greatest barriers to uptake in this study [27].

In the study in Burkina Faso by Schlumberger et al., the delay for vaccination study was also done, and has shown similar results to the study by Haji and Bangure, significantly more timely vaccinations in the intervention group. The study also attempted to find the reasons for delayed or missed vaccinations in both intervention and control groups. Using telephone call back, the study found that 19% of the phone numbers called were disabled or now being used by a new owner with no association to the mother or the child. Of the answered calls, the reasons for missing the vaccination were shown to be due mostly to: children attending another health center (66%), children travelling with parents (18%), and parents’ negligence (13%) [28].

In the study in Senegal, the timeliness of the Vitamin supplement intake was also measured, and found that the average age of supplementation for children pre-intervention is much older than post-intervention with SMS reminders. The study also included an SMS stock reporting for Vitamin A supplements availability in health posts. The stock report helped ensure that 100% of health posts had stock of Vitamin A capsules throughout the intervention period, with all intervention districts succeeding in submitting at least 80% of their weekly stock reports. The comparison was not made however, with the stock availability before the intervention, since earlier, Vitamin A stocks were distributed only twice per year and was not routinely given out. Since the communities in the study has very low initial knowledge of Vitamin A supplementation, which led to low supplementation rate, a questionnaire was also utilized to find how the mothers learn about the Vitamin A supplementation. The questionnaire revealed that before the intervention, more than 80% of the sources of information came from communications by health workers and radio. However, after intervention, the two intervention districts showed that 78% and 76% of sources of supplementation for the mothers were phone call and SMS. Social mobilization or communications by health workers became a much smaller source of information (10-15%) for intervention districts, showing that one could get high supplementation coverage even with less use of human resources, and that SMS messages or phone calls can replace traditional communication method to a great effect [23].

### SMS alerts during immunization campaigns in Kenya

In the 2016 SIA, a research led by the Ministry of Health and in collaboration with Kenya Red Cross and US Centers for Disease Control, was done in Kenya’s Western region to explore the feasibility of using the mobile phones to improve immunization nationwide. The study sent multiple bulks of SMS reminder messages about the MR campaign (including messages informing the dates, locations, target group for vaccination, and importance of vaccine) through the Safaricom and Airtel networks in selected counties [29].

## Discussion

From the seven studies done on SMS vaccine reminders in Africa, all have shown marked improvements in the different metrics for vaccination, whether it is through the increase in vaccination coverage (Gibson et al., Bangure et al., Thiaw et al.), decrease in dropout rates (Haji et al.), increase in completion rate (Brown et. al., Eze, Schlumberger et al.), or decrease in delay for vaccination (Bangure et al., Haji et al., Schlumberger et. al.).

In terms of the cost effectiveness of SMS as vaccine reminder, Eze has shown the cost per children for using SMS reminder is much lower than home visit, a current method of vaccination reminders in many African countries. The biggest cost reduction that was made in that study were salaries of workers and logistics, both massive costs in the house-to-house campaigns [30]. Although the equipment and internet service are two of the biggest additional costs, they can be used long-term and provide ownership of the system to the workers themselves. This way, the workers can manage their health areas and increase sustainability in the system. A study by Thiaw et al. further showed that when comparing the intervention group, where SMS and phone call reminders were the main source of information, with the control group, where social mobilization and health worker communication are the main source of information on Vitamin A supplement, the uptake of Vitamin A is still greater in the intervention group [23]. This shows that the use of health worker is not necessary for the uptake of the supplements, and that SMS or phone technology can replace health workers as a source of information and also produce better uptake results.

Literacy was shown to be an issue in SMS reminder system in two studies, and must be addressed when SMS-reminder system is being planned for implementation. A few suggestions include keeping the message simple, using automatic voice messages or calls, or instructing the mothers to ask a literate community member to explain the message [30]. Another issue that the studies faced frequently was the mobile phone number change, which causes the investigator to lose track of the registered mother [31]. In Africa, phone number changes happen frequently due to unreliability of the telecommunication services. Therefore, the SMS-reminder system must have a built-in system for mothers to update their phone numbers.

Other than forgetfulness and negligence, the biggest factors to missing vaccination, as founded in the studies, were being in the middle of travel, changing health facility and getting off the investigator’s tracking system, and distance from health facility. One must look to design a system that addresses these identified challenges. One way to help remind mothers who are travelling, is to have a recall system that some of the studies used, in order to keep reminding the mother until she is available to take her child to be vaccinated. To combat changing of health facilities, one must design a system that covers all health facilities and connects their patients’ data together in one registration platform. The distance from health facility is an important issue and one that unfortunately cannot be solved simply through an improved SMS system only.

In terms of the systems used, all studies used different software to create this SMS reminder platform for immunization. To create a sustainable and scalable system, one must look to develop the program from existing and accepted products. Rapid SMS, a software developed by UNICEF, is an example of a software that is flexible, and built as an open-sourced software. Rapid SMS and its successors, RapidPro and Textit, could become a more common foundation to build a system on in the future.

## Conclusion

Although the studies reviewed showed that SMS reminder system is undisputedly a highly qualified tool for increasing vaccination uptake, the system itself is not the sole factor to be considered when trying to improve vaccination status. Literacy, movements of the population, health facilities, and remoteness of the area all play a part to affect vaccination of the population. Policy efforts should be put to address these areas where technology can only play a minor role. However, as it stands, SMS reminder for vaccination has proven to be effective in multiple control trials and pilot studies done in Africa. Through this review, one can conclude that a full-scale implementation of this system should be the next step for African countries to fight the low vaccination coverage in this region.

### What is known about this topic

- One of the biggest reasons for missed vaccination in children in Africa is the lack of communication between mothers and health workers, which could lead to a lack of knowledge on vaccination, missing the vaccination schedule, or lack of proper service or lack of vaccines on the day of vaccination;
- Mobile phones are becoming more accessible in Africa, as the costs of services are getting cheaper; ownership has been surging rapidly, with some countries achieving more than ten-fold increase in ownership from 2002 to 2014;
- Overall, more than 40 published studies in Cochrane review from different regions have proven the effectiveness of vaccine reminders, although no study has been done to review all the studies for SMS-based vaccine reminders in Africa.

### What this study adds

- The study provides detail into the methods of 6 different studies in Africa which use SMS vaccine reminder system, and could aid any people who want to develop the system to understand;
- The study contains both unpublished articles, articles from grey literature, and reports that were not published, in order to provide a comprehensive view on the uses of this system;
- The study looks into the details of the SMS system, which could give a good starting point for any individuals, organizations, or companies that are looking to create an SMS vaccine reminder system.

## Competing interests

The authors declare no competing interests.

## References

[1] Anderson RM, May RM (1985). Vaccination and herd immunity to infectious diseases. Nature..

[2] World Health Assembly (27th: 1974) (1985). The Expanded Programme on Immunization: the 1974 Resolution by the World Health Assembly. Assignment Child..

[3] WHO (2016). Fact Sheet. Immunization Coverage.

[4] WHO (2016). Immunization, Vaccines and Biologicals. Global immunization coverage sustained in the past five years.

[5] WHO (2016). Fact Sheet. Immunization Coverage.

[6] WHO (2016). Fact Sheet. Immunization Coverage.

[7] Expanded Programme on Immunization (EPI) in the African Region (2001). strategic plan of action 2001-2005.

[8] WHO (2015). Immunization, Vaccines and Biologicals. Updated data on immunization coverage published by WHO and UNICEF.

[9] WHO. AFRO (2016). Despite gains in access, 1 in 5 African children go without lifesaving.

[10] Task Force on Community Preventive Services (1999). Vaccine-preventable diseases: improving vaccination coverage in children, adolescents, and adults. MMWR- Morbidity & Mortality Weekly Report..

[11] Jacobson Vann JC, Szilagyi P (2005). Patient reminder and recall systems to improve immunization rates. Cochrane Database of Systematic Reviews.

[12] Hersman E (2013). The Mobile Continent. Stanford Social Innovation Review 10th Anniversary Essays. Spring.

[13] Ericsson (2014). Sub-Saharan Africa. Ericsson Mobility Report Appendix.

[14] Favin M, Steinglass R, Fields R, Banerjee K, Sawhney M (2012). Why children are not vaccinated: a review of the grey literature. International Health..

[15] Jani JV, De Schacht C, Jani IV, Bjune G (2008). Risk factors for incomplete vaccination and missed opportunity for immunization in rural Mozambique. BMC Public Health.

[16] Health Canada (2004). Measuring up: Results from the national immunization coverage survey, 2002. Canada Communicable Disease Report.

[17] Abdulraheem IS, Onajole AT, Jimoh AAG, Oladipo AR (2011). Academic Journals Full Length Research Paper Reasons for incomplete vaccination and factors for missed opportunities children. Journal of Public Health and Epidemiology.

[18] Glaxo Smith Kline Behind the Science (2016). The power of partnerships: transforming vaccine coverage in Mozambique.

[19] Chiu K, Spigel L (2016). Study of the effectiveness of strategies and methods for providing immunization reminders to parents and guardians of children in Benin. VaxTrac BID Initiative.

[20] Gibson D (2016). The Mobile Solutions for Immunization (M-SIMU) Trial: a protocol for a cluster randomized controlled trial that assesses the impact of mobile phone delivered reminders and travel subsidies to improve childhood immunization coverage rates and timeliness in Western Kenya. JMIR Res Protoc..

[21] Brown VB, Oluwatosin OA, Akinyemi JO, Adeyemo AA (2016). Effects of Community Health Nurse-Led Intervention on childhood routine immunization completion in primary health care centers in Ibadan, Nigeria. J Community Health..

[22] Haji A, Lowther S, Ngan’ga Z (2016). Reducing routine vaccination dropout rates: evaluating two interventions in three Kenyan districts, 2014. BMC Public Health.

[23] Thiaw C, Cooper A, Beye M, Tendeng C, Thiam K, Thiam A (2012). Routine Delivery of Vitamin A Supplementation at Six Months in Senegal Using SMS Reminder Messages: Results from the Pilot of Routine Delivery of Vitamin A Supplementation at 6 Months..

[24] Bangure D, Chirundu D, Gombe N, Marufu T, Mandozana G, Tshimanga M (2015). Effectiveness of short message services reminder on childhood immunization programme in Kadoma, Zimbabwe - a randomized controlled trial, 2013. BMC Public Health..

[25] Kebede M, Zeleke A, Asemahagn M, Fritz F (2015). Willingness to receive text message medication reminders among patients on antiretroviral treatment in North West Ethiopia:a cross-sectional study. BMC Med Inform Decis Mak..

[26] Haji A, Lowther S, Ngan’ga Z (2016). Reducing routine vaccination dropout rates: evaluating two interventions in three Kenyan districts, 2014. BMC Public Health..

[27] Eze GU, Adeleye OO (2015). Enhancing Routine Immunization Performance using Innovative Technology in an Urban Area of Nigeria. West Afr J Med..

[28] Schlumberger M, Bamoko A, Yameoogo TM, Rouvet F, Ouedraogo R (2015). Positive impact on the Expanded Program on Immunization when sending call-back SMS through a Computerized Immunization Register, Bobo Dioulasso (Burkina Faso). Bull Soc Pathol Exot..

[29] Scobie H Personal Communications. From pilot study by Kenya Ministry of Health, in collaboration with Kenya Red Cross and support from the US Centers for Disease Control..

[30] Eze GU, Adeleye OO (2015). Enhancing Routine Immunization Performance using Innovative Technology in an Urban Area of Nigeria. West Afr J Med..

[31] Schlumberger M, Bamoko A, Yameoogo TM, Rouvet F, Ouedraogo R (2015). Positive impact on the Expanded Program on Immunization when sending call-back SMS through a Computerized Immunization Register, Bobo Dioulasso (Burkina Faso). Bull Soc Pathol Exot..

